# Letter from Dr. Kenneth Wilson

**DOI:** 10.3390/curroncol13020007

**Published:** 2006-04

**Authors:** Kenneth S. Wilson

**Affiliations:** Division of Medical Oncology, BCCA Vancouver Island Centre, 2410 Lee Avenue, Victoria V8R 6V5

**Re:** Systemic therapy for patients at high risk for recurrent melanoma. Verma S., Quirt I., McCready D., Charette M., Iscoe N. and the members of the Melanoma Disease Site Group. *Curr Oncol* 2005; 12:31–6.

The conclusions reached in this review are based on incomplete data. In June 2002, the Melanoma Disease Site Group (dsg) sent me a draft manuscript of the above paper to review. I specifically recommended that the proposed guideline should reflect the fact that the E1684 trial is *negative* for overall survival benefit for high-dose interferon (hdifn) at a median follow-up of 12.6 years (one-sided *p* value 0.09, as dictated by the protocol). Dr. John Kirkwood, principal investigator of the E1684 trial, provided a *p* value for overall survival in a letter to *J Clin Oncol* 2001 in response to a letter of mine seeking same. Unfortunately, Dr. Kirkwood, like the dsg, continues to interpret E1684 as a positive study, in spite of the insignificant *p* value with mature follow-up*. Although the E1684 investigators point to competing causes of death, they have not analyzed disease-specific mortality—or if they have, they have not reported it. They have reported analysis of distant disease-free survival, but this was not a protocol-specified endpoint. During manuscript review, I recommended that the dsg obtain the 12.6-year follow-up data of E1684 to add to their database and conduct joint analysis with other ecog trials. The dsg have not acknowledged this important follow-up data on E1684 in their guideline. In fact, they reiterate that the ecog 1684 trial detected a significant improvement in overall survival after “prolonged follow-up.” This was in spite of being advised that analysis at a median follow-up of 12.6 years is negative (*p* = 0.09) for overall survival benefit. Consequently, we now have two mature ecog studies of adjuvant hdifn that are negative for overall survival benefit.

I believe that the results of E1694 are too preliminary to conclude that hdifn is superior to gmk vaccine as adjuvant therapy of high-risk melanoma. The importance of mature follow-up is amply demonstrated by the E1684 experience. Indeed, the absence of published follow-up data on the E1694 trial makes one wonder whether the preliminary results are sustained.

## Footnotes

* Wilson K. High-dose interferon versus GM2 vaccine in high-risk malignant melanoma [comment/reply]. *J Clin Oncol* 2001;19:4350.

